# Cpx-signalling in *Yersinia pseudotuberculosis* modulates Lipid-A remodelling and resistance to last-resort antimicrobials

**DOI:** 10.1038/s44259-024-00059-y

**Published:** 2024-11-18

**Authors:** Dharmender K. Gahlot, Jonasz B. Patkowski, Jaime Fernández de Santaella, Luke P. Allsopp, Zhiqiao Pan, Alain Filloux, Gerald Larrouy-Maumus, Matthew S. Francis, Tiago R. D. Costa

**Affiliations:** 1 Department of Molecular Biology and Umeå Centre for Microbial Research (UCMR), Umeå University, Umeå, Sweden; 2 Centre for Bacterial Resistance Biology, Department of Life Sciences, Imperial College London, London, UK; 3 National Heart and Lung Institute, Imperial College London, London, UK; 4 School of Biological Sciences, Nanyang Technological University Singapore, 637551, Singapore; 5 Lee Kong Chian School of Medicine, Nanyang Technological University, Singapore, Singapore

**Keywords:** Antibacterial drug resistance, Bacterial genetics, Lipopolysaccharides

## Abstract

Antibiotic resistance is a global healthcare crisis. Bacteria are highly adaptable and can rapidly acquire mechanisms of resistance towards conventional antibiotics. The permeability barrier conferred by the Gram-negative bacteria cell envelope constitutes a first line of defence against the action of antibiotics. Exposure to extracytoplasmic stresses can negatively affect cell envelope homoeostasis and this causes localised protein misfolding, compromised envelope integrity and impairs barrier function. The CpxA-CpxR two-component regulatory system has evolved to sense extracytoplasmic stresses and to regulate processes that restore homoeostasis of the cell envelope. Hence, controlled Cpx-signalling assists bacteria in adapting, surviving and proliferating in harsh environments, including exposure to antibiotics. Herein, we determined that an intact Cpx-signalling is key to maintaining the *Yersinia pseudotuberculosis* resistance to colistin and polymyxin B. The susceptibility displayed by Cpx-signalling defective mutants, correlated with cell-envelope deformity and specific modifications of Lipid-A. In vivo transcriptional analysis and in vitro protein-DNA binding studies demonstrated that these modifications were dependent on the direct regulation of Lipid-A biogenesis and modifications of operons by the active phosphorylated CpxR~P isoform. Altogether, our work defines the regulatory mechanism that enables Cpx-signalling to actively control cell envelope remodelling and the permeability of antibiotics in the clinically relevant enteropathogen *Y. pseudotuberculosis*.

## Introduction

The World Health Organisation labels antibiotic resistance as a global health crisis that requires immediate multisectoral action to achieve sustainable healthcare^1^. Inadequate discovery of new antibiotics has focused attention on the research for additional drug targets in bacteria and understanding the mechanisms of action and resistance to the ones we currently have. Polymyxin E, also known as colistin, is a last-resort broad-spectrum antibiotic used either alone or in combination with other antimicrobials to treat infections caused by multi-drug resistant Gram-negative *Enterobacteriaceae* bacteria where β-lactam, aminoglycoside or quinolone antibiotics are ineffective^2,3^. The related polymyxin B, that differs from colistin by a single-amino-acid (phenylalanine instead of leucine in colistin) in the cyclic heptapeptide part of the chemicals, is also in current clinical use^4^. Yet, misuse of colistin and polymyxin B has culminated in a rise of bacteria resistant to these agents^5^. Hence, resolving the issue of multi-antibiotic-resistant bacterial infections ultimately needs the development of new drugs against targets that do not lead to the rapid emergence of drug resistance^6^.

The permeability barrier function of the bacterial cell wall is an intrinsic resistance mechanism in bacteria^7^. In Gram-negative bacteria, the outer membrane is a key component regulating the influx of a range of harmful compounds including antibiotics^8,9^. A typical outer membrane is an asymmetrical lipid bilayer. The outer leaflet is almost exclusively composed of glycolipids and the inner leaflet of phospholipids^8^. Glycolipids in the outer leaflet are either attached to long repeats of sugar moieties and constitute lipopolysaccharide or are connected to a short oligosaccharide to form lipooligosaccharide^8^. How these lipids are assembled into a continuous barrier, and how this barrier is preserved in response to damage or extracellular stress remain fascinating fundamental biological questions^10,11^. The outer membrane also possesses lipoproteins and β-barrel porins or channel proteins^8,12^. Channel proteins facilitate cellular permeability and are another crucial element to supporting membrane integrity and facilitating barrier function^12–15^.

Disruption of the barrier function is induced by various extra cytoplasmic stresses, which leads to an increased susceptibility to antimicrobials^16^. To preserve barrier function, bacteria express certain sigma (σ) factors^17^ and two-component signal transduction (TCST) systems^18^ as sentinels to sense and coordinate responses to the stress insults. TCST systems function through histidine-aspartate phosphorelay involving at least two components—a histidine protein kinase and a response regulator. Histidine protein kinases are typically composed of an input domain that senses environmental conditions and a transmitter domain that auto-phosphorylates on a histidine residue in an input-sensitive manner. Response regulators are composed of a receiver domain that transfers the high-energy phosphoryl group from the histidine protein kinase to an aspartate residue of its own, and an output domain the function of which is usually to activate or repress transcription depending upon the phosphorylation state of the receiver. The classical CpxAR TCST system is present in a large family of Gram-negative bacteria. It responds to protein mislocalisation or misfolding in the cell envelope primarily caused by external osmolarity, pH, ethanol, copper, detergent, and EDTA-induced stresses^19,20^. CpxA is a dual functional histidine kinase and phosphatase sensory protein, located in the inner membrane, whereas CpxR is the cognate cytoplasmic response regulator^21,22^. Critically, the CpxAR system coordinates quality control of the bacterial envelope with the maintenance of fitness for survival in the environment and during host infections. In fact, virulence gene expression is under the control of CpxAR systems in numerous clinically relevant bacterial pathogens^23–31^. The enteropathogen *Yersinia pseudotuberculosis* has long served as a relevant model for pathophysiological studies of bacterial pathogens. In this pathogen, the CpxAR system contributes to survival in stressful environments by regulating the global regulators, RovA^32^ and RovM^33^, as well as coordinating bacterial adhesion^34^, biofilm formation^30^ and protein secretion^35^.

Interestingly, an association between CpxAR and RpoE (σ^E^) sigma factor contributes towards restoration of cell envelope integrity under aerobic and phosphate starvation conditions, and this protects non-growing *Escherichia coli* from gentamicin killing^36^. Furthermore, coupling between CpxAR and the PmrAB and PhoPQ TCST systems reportedly mediates the susceptibility of *Salmonella enterica* serovar Typhimurium to colistin^37^. Moreover, multi-drug resistance to amino-glycoside and β-lactam antibiotics by *S. enterica* serovar Typhimurium may also work through CpxAR signalling^38^. Significantly, the mechanism whereby CpxAR controls colistin, amino-glycoside and β-lactam antibiotics susceptibility in bacteria is unknown. Hence, achieving a comprehensive understanding of the contribution of the CpxAR system to preserve membrane barrier function and antibiotic resistance in prevalent clinical pathogens may define new molecular targets for novel antibacterial drug development.

Therefore, in this study, we explored the contribution of Cpx-signalling in membrane remodelling and antibiotic susceptibility using *Y. pseudotuberculosis* strain YPIII/pIB102 (*Yptb*-YPIII). Studying this experimental system is of interest because it exhibits a characteristic temperature-dependent biphasic environmental and infectious shift—a trait common to many pathogens, which impacts on virulence, membrane integrity and antibiotic resistance profiles of *Yersinia* through Lipid-A modifications^39–41^. We started by probing the bacterial envelope integrity from parental and Δ*cpxA* mutant bacteria with colistin, observing that Cpx-signalling defective mutants displayed increased sensitivity to colistin and polymyxin B. The construction details for mutant generation together with an initial phenotypic characterisation were described previously^35,42^. Increased sensitivity correlated with specific CpxA-dependent modifications of Lipid-A, a core constituent of LPS in the bacterial outer membrane. These modifications were brought about through a direct transcriptional regulation of the Lipid-A biosynthetic and/or modification operons, *arnABCD, lpxPL* and *pmrE*. Hence, this study defines new critical components of the Cpx-regulon that enables Cpx-signalling to actively regulate permeability of antimicrobials including the last-resort broad-spectrum antibiotics, colistin and polymyxin B.

## Results

### Accumulation of active CpxR ~ P isoform increases *Yersinia* susceptibility to colistin

**Fig. 1:**
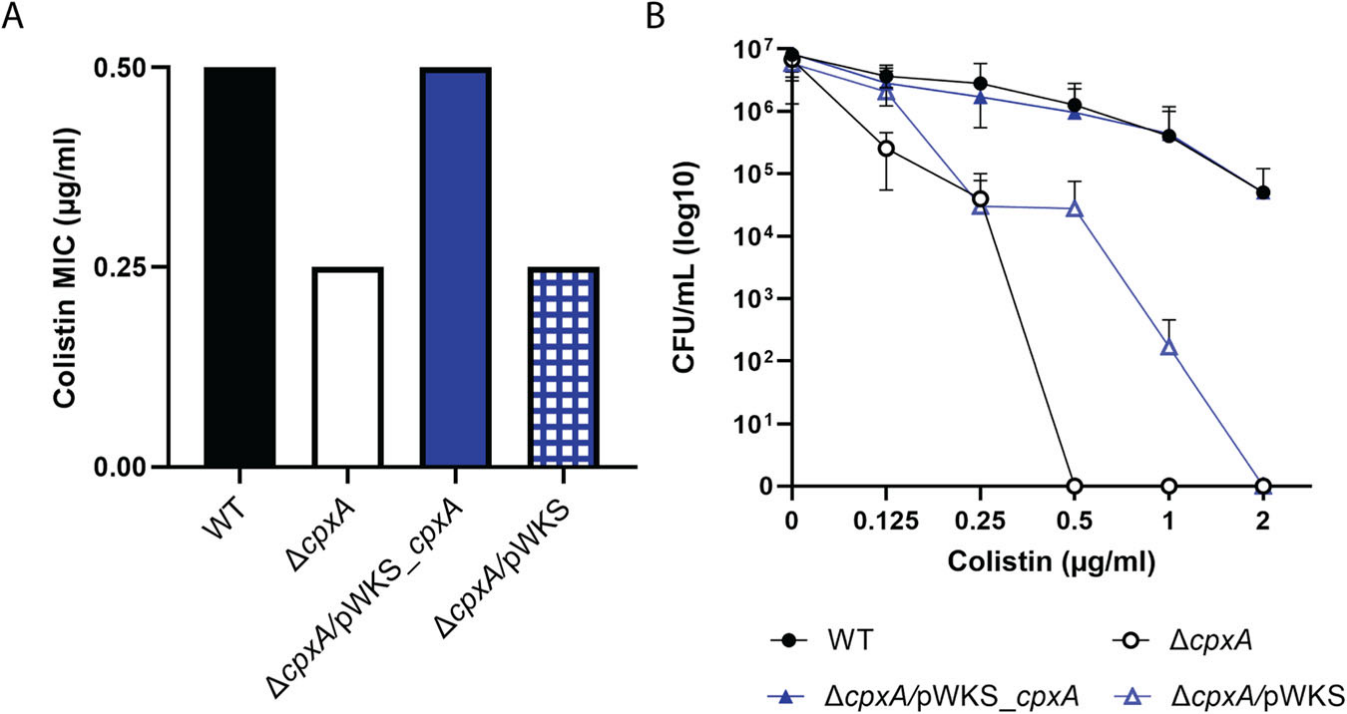
Loss of CpxA renders *Yersinia pseudotuberculosis* more susceptible to colistin which can be rescued via complementation. **A** Minimal inhibitory concentrations (MIC) to colistin of parental (WT), Δ*cpxA*, Δ*cpxA****/***pWKS_*cpxA*, and Δ*cpxA*/pWKS strains. Representative of at least three separate experiments. **B** 1-h colistin time-kill assay plotted as colony forming units (CFU) shows enhanced killing of Δ*cpxA* and Δ*cpxA*/pWKS strains compared to parental (WT) or Δ*cpxA****/***pWKS_*cpxA*. The difference between parental (WT) or complemented (Δ*cpxA****/***pWKS_*cpxA*) against Δ*cpxA* or Δ*cpxA****/***pWKS is statistically significant (for example concentration 0.5, *P* < 0.05, two-way ANOVA Tukey’s multiple comparison test). Graph depicts data from three biological experiments.

To investigate the contribution of Cpx-signalling in *Yptb*-YPIII toward colistin susceptibility, we first examined whether accumulation of the active CpxR~P isoform affected susceptibility to the last-resort antibiotic, colistin. We used two isogenic strains of YPIII (*Yptb*-YPIII); the first is a clinical isolate that is naturally PhoP-defective^43^ that for the purpose of this study is designated as the wild-type (WT) isolate because it contains an intact CpxAR system that when unstimulated maintains very low levels of active CpxR~P isoform (Supplementary Fig. S1), and the second was the Δ*cpxA* null mutant that lacks the negative feedback loop reliant upon phosphatase activity and thereby permits constitutive production of active CpxR~P isoform that accumulates in the cytoplasm (Supplementary Fig. S1)^30^. Hence, we study the effects of Cpx-signalling in isolation from the PhoPQ system. A comparison of the susceptibility to colistin or polymyxin B was accomplished by monitoring bacterial growth in media containing sequential increases in the concentration of the antibiotics. We determined the minimal inhibitory concentration (MIC) of colistin to be 0.5 μg/ml for the parental (WT) and 0.25 μg/ml for the Δ*cpxA* mutant (Fig. 1A). Although only a 2-fold difference, we validated our findings through complementing of Δ*cpxA* with pWKS_*cpxA* which restored the MIC to 0.5 μg/ml, whereas this was not the case for the vector control pWKS (Fig. 1A). The expression of *cpxA* in pWKS is regulated by the native *cpxRA* promoter. Construction details of the vector have been described elsewhere^33^.

The observed differences in colistin susceptibility between the strains was not explained by deviations in their normal growth profiles since all strains grew comparatively (Supplementary Fig. S2). A one-hour time kill curve showed that with increasing concentrations of antibiotics fewer CFUs could be recovered as expected for colistin treatment, but this was especially apparent for the Δ*cpxA* with Δ*cpxA*/pWKS strains in line with the above findings (Fig. 1B). To probe this susceptibility profile further we repeated these assays with polymyxin B and observed comparative drops in the MIC and significantly faster killing of Δ*cpxA* with Δ*cpxA*/pWKS strains at one hour (Supplementary Fig. S3AB). Hence, accumulation of active CpxR~P isoform enhances susceptibility to colistin and polymyxin B in enteropathogenic *Yptb*-YPIII, corroborating recent findings in other bacteria^37,44^.

### Increased colistin susceptibility correlates to changes in Lipid-A acylation and glycosylation

**Fig. 2:**
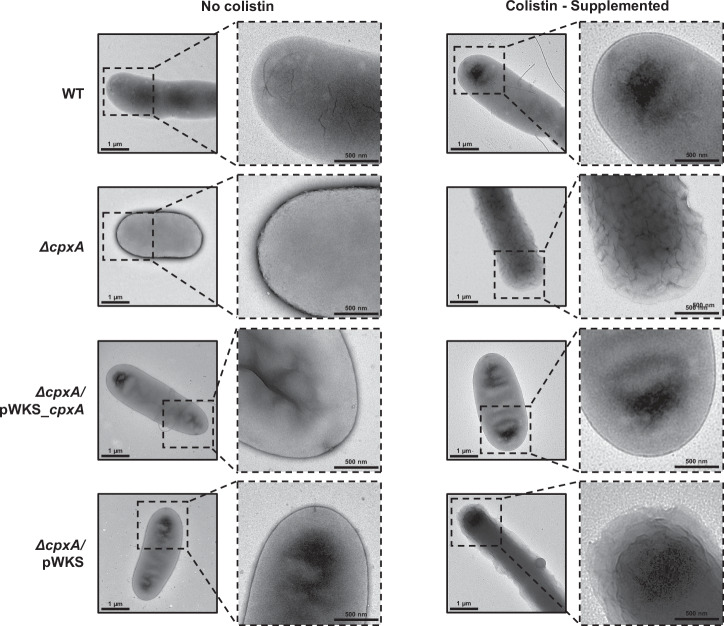
Colistin-induced changes in the cell envelope of CpxA defective *Y. pseudotuberculosis.* Representative transmission electron micrographs of *Y. pseudotuberculosis* parent (WT), Δ*cpxA*, Δ*cpxA/*pWKS_*cpxA* complementation strain and an empty plasmid control Δ*cpxA/*pWKS bacteria were cultured with and without, 2 μg/ml of colistin for 30 min at 26 °C.

Given that the CpxAR system maintains bacterial envelope integrity^45–47^, it is anticipated that Cpx-dependent modulation of colistin susceptibility would involve changes in cell envelope barrier function. To gain an understanding of the molecular mechanism for increased colistin susceptibility of *Yptb-*YPIII, we used transmission electron microscopy to visualise the bacterial cell surface after a 30-minute incubation with or without colistin of an initial culture grown to an OD_600_ = 1.0. We observed dramatic structural changes to the cell membrane of the Δ*cpxA* mutant when bacteria were grown in the presence of colistin (Fig. 2). These structural distortions were not evident in parental (WT) bacteria where Cpx-signalling remains intact (Fig. 2). It has been demonstrated that absence of CpxA leads to an accumulation of phosphorylated CpxR (Fig. S1) because CpxR can be phosphorylated via alternative pathways^27,32,48–50^. This accumulation has been linked to many changes in the bacterial envelope, including alterations in peptidoglycan and protein content, and any such change could result in bacterial surface alterations visible by electron microscopy upon treatment with colistin. Notably, some cells appear longer than others. However, this is unlikely to be related to the treatment with colistin, but rather due to different stages of bacterial growth^51^.

**Fig. 3:**
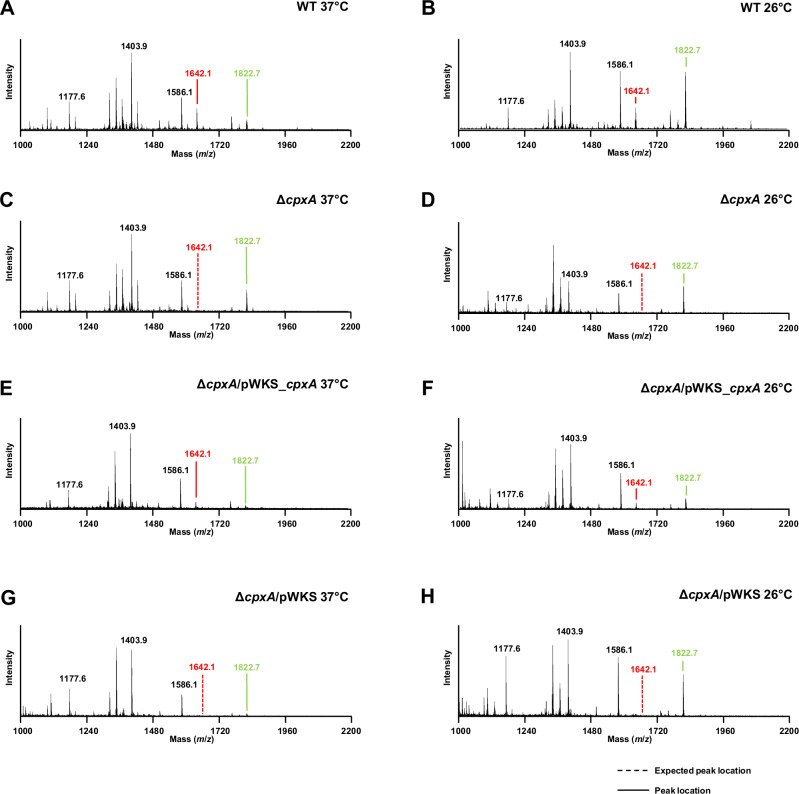
Absence of CpxA induces Lipid-A modifications. Representative mass spectra of Lipid-A from intact parent (WT) bacteria grown at 37 °C (**A**) and 26 °C (**B**), Δ*cpxA* bacteria grown at 37 °C (**C**) and 26 °C (**D**), Δ*cpxA* complementation strain Δ*cpxA/*pWKS_*cpxA* grown at 37 °C (**E**) and 26 °C (**F**) as well as an empty plasmid control Δ*cpxA/*pWKS grown at 37 °C (**G**) and 26 °C (**H**). Spectra was acquired using matrix-assisted laser desorption ionization (MALDI). A set of 5 peaks was identified as unique Lipid-A species and their respective chemical classifications are provided in Table 1. The peak at *m/z* 1642.3 is identified as a 4-amino-L-arabinose (L-Ara4N) modification of a penta-acylated Lipid-A (red), and the peak at *m/z* 1822.7 as hexa-acylated Lipid-A (green). Dashed line represents the expected location of a peak that is not present.

To investigate the molecular and biochemical basis for this observation, we focused on the outer leaflet of the outer membrane, as this is the first component contributing to the impermeability of hydrophobic molecules such as colistin^5^. Lipid-A species from total preparations of LPS were isolated from both parental (WT) and Δ*cpxA* mutant, as well as Δ*cpxA/*pWKS_*cpxA* complementing strain and Δ*cpxA/*pWKS empty plasmid control. All bacteria were grown until mid-logarithmic phase at either 26 °C or 37 °C. We used two growth temperatures because *Yptb* displays a biphasic environmental (26 °C) and infectious lifecycle (37 °C) where thermoregulation represents an important element of gene expression reprogramming to facilitate bacterial transitioning from one phase to the other^52,53^. Several isolated Lipid-A species were detected by MALDI-TOF (Fig. 3 and Table 1). Five discriminatory Lipid-A peaks were labelled (Fig. 3) and their structural assignments provided in Table 1. The peak at *m/z* 1642.2 was present in all parental (Fig. 3A, B) and complementing Δ*cpxA/*pWKS_*cpxA* **(**Figs. 3E and 3F**)** strains grown at the two different temperatures but absent in Δ*cpxA* mutant strains (Fig. 3C, D) or empty plasmid control strain Δ*cpxA/*pWKS **(**Fig. 3G and 3H**)**. This clear difference indicates the importance of an intact CpxRA TCST system for the incorporation of 4-amino-L-arabinose (L-Ara4N) modifications in penta-acylated Lipid-A, potentially through CpxR~P accumulation. On the other hand, the *m/z* 1822.7 peak that corresponds to hexa-acylated Lipid-A was present in both temperatures, regardless of CpxA expression. Together, these data indicate that accumulation of CpxR~P has an influence on the regulation of genes that control some Lipid-A modifications.

**Table 1:** Characterised Lipid-A species detected and their corresponding structural assignment

Theoretical ion (m/z)	HPO_3_	Ara4N	Predicted Structure	Name
**1177.6**	2	0	3xC14:0, 2xP	3 x myristic acid, 2 x phosphate
**1403.9**	2	0	4xC14:0, 2xP	4 x myristic acid, 2 x phosphate
**1586.1**	2	0	4xC14:0, C12:0, 2xP	4 x myristic acid, lauric acid, 2 x phosphate
**1642.2**	2	1	4xC14:0, C16:1, 2xP, Ara4N	4 x myristic acid, palmitoleic acid, 2 x phosphate, aminoarabinose
**1822.7**	2	0	C12:0, 4xC14:0, C16:1, 2xP	lauric acid, 4 x myristic acid, palmitoleic acid, 2 x phosphate

### Transcriptional regulation of Lipid-A acylation and glycosylation pathways by active CpxR ~ P

**Fig. 4:**
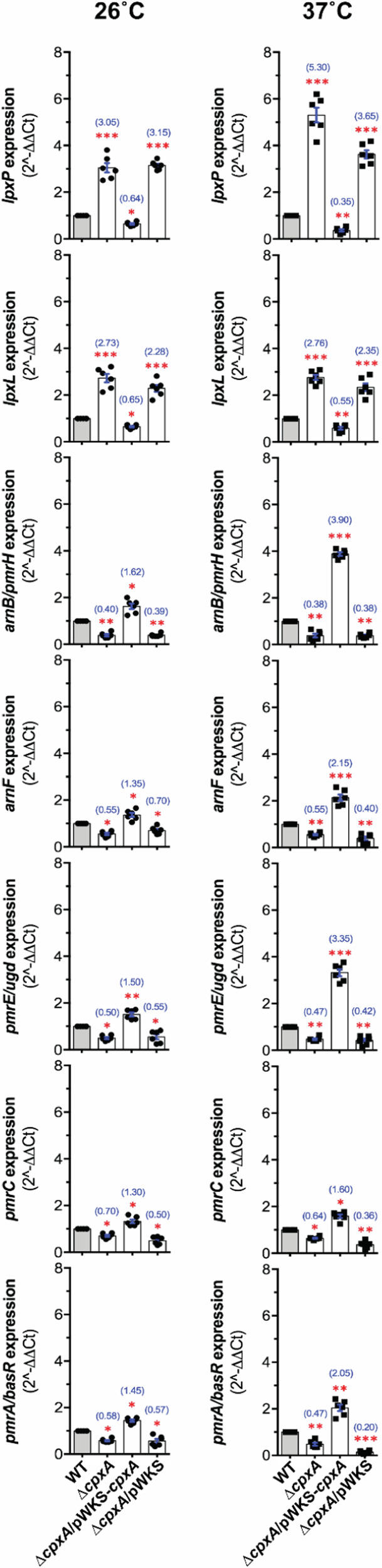
Gene specific transcription is altered by loss of CpxA in *Y. pseudotuberculosis.* Quantitative RT-PCR was performed on mRNA isolated from *Y. pseudotuberculosis* strain YPIII07/pIB102 (Δ*cpxA* full-length null mutant) cultured in LB at 26 °C (**A**) or at 37 °C (**B**) until mid to late logarithmic phase (*i.e*.: an incubation period of 5 h). Data is a collation of four independent experiments composed of three technical replicates in which gene specific transcription of the 12 values was separately normalised against the mean cycle threshold of three independent housekeeping genes, 16S rRNA gene (*n* = 12), *gyrB* (*n* = 12), and *rpoC* (*n* = 12). After which all 36 data values are individually represented in a scatter plot as fold change relative to the calibrator control (parental *Y. pseudotuberculosis* strain YPIII/pIB102 normalised to Log_2_fold = 0). The median value from all data points is indicated by a blue line, and the actual fold difference in up- (>1.0) or down-regulation (<1.0) of specific gene expression relative to calibrator is indicated in parenthesis. Statistical analysis was performed using a two-tailed, unpaired t-test with Welch’s correction where equal variance is not assumed, and confidence interval was set to 99%. Differences with a *P*-value of <0.05, < 0.01 or < 0.001 were considered significantly different from parent and are indicated by a red single (*), double (**) or triple (***) asterisk situated immediately above the respective data points on the scatter plot. Analysed genes were: *lpxP* encoding for Palmitoleoyl acyltransferase (locus tag YPK_0430); *lpxL* encoding for Lauroyl acyltransferase (YPK_1665); *arnB/pmrH/L-ara4N* encoding for UDP-4-amino-4-deoxy-L-arabinose-oxoglutarate aminotransferase (YPK_1831); *arnF* encoding for Undecaprenyl phosphate-alpha-L-ara4N flippase subunit ArnF (YPK_1837); *pmrE/ugd* encoding for UDP glucose 6-dehydrogenase (YPK_2077); *pmrC* encoding for serine-type D-Ala-D-Ala carboxypeptidase/endopeptidase (penicillin-binding protein 4) (YPK_3740); *pmrA/basR* encoding for the two-component response regulator PmrA (YPK_3741).

In line with the CpxAR system coordinating several important processes in *Yptb-*YPIII^30,32–35^, we hypothesised that active phosphorylated CpxR~P acts as a transcription factor to regulate Lipid-A modification pathways in this bacterial pathogen. To investigate this, we first sought to confirm under the same assay conditions employed above that the active phosphorylated form of CpxR~P accumulated in the Δ*cpxA* mutant. When analysed by the Phos-tag^TM^ acrylamide gel system it was evident that protein lysate material contained active phosphorylatable CpxR~P accumulated to a high level in the *cpxA* null-mutant and this mutant harboring the empty vector pWKS control, but this was reduced to parental levels in the mutant complemented with pWKS_*cpxA* (Supplementary Fig. S1A). We used an isogenic mutant encoding *cpxR*_D8A, D9A, D51A, M53A, K100A_ that only produces a non-phosphorylated CpxR (CpxR_Pneg_) to confirm the specificity of this assay (Supplementary Fig. S1B). To investigate if this accumulation of active CpxR~P correlated with alterations in gene expression within the Lipid-A acylation and glycosylation operons in *Yptb*-YPIII, we examined the transcriptional profile of seven target genes: *lpxP* (YPK_0430), encoding for Palmitoleoyl acyltransferase; *lpxL* (YPK_1665), encoding for Lauroyl acyltransferase; *arnB/pmrH/L-ara4N* (YPK_1831), encoding for UDP-4-amino-4-deoxy-L-arabinose-oxoglutarate aminotransferase; *arnF* (YPK_1837), encoding for Undecaprenyl phosphate-alpha-L-ara4N flippase subunit, ArnF; *pmrE/ugd* (YPK_2077), encoding for UDP glucose 6-dehydrogenase; *pmrC* (YPK_3740), encoding for serine-type D-Ala-D-Ala carboxypeptidase/endopeptidase (penicillin-binding protein 4); and *pmrA/basR* (YPK_3741), encoding for the two-component response regulator, PmrA. Operon structures of Lipid-A acylation genes, *lpxP* (YPK_0430) and *lpxL* (YPK_1665) are indicated in Supplementary Fig. S4 and operon structures of Lipid-A glycosylation genes, *arnB/pmrH/L-ara4N* (YPK_1831), *pmrE/ugd* (YPK_2077) and *pmrC* (YPK_3740) are shown in Supplementary Fig. S5. Using qRT-PCR, endogenous transcriptional expression of these genes was measured at mid to late logarithmic phase of growth in the constitutive active CpxR~P expressing mutant strain, Δ*cpxA* compared to the parental (WT) strain at 26 °C (Fig. 4A) and at 37 °C (Fig. 4B). Compared to parental (WT) bacteria, active CpxR~P (in Δ*cpxA*) significantly elevated the expression of both Lipid-A acylation genes, *lpxP* (YPK_430) and *lpxL* (YPK_1665) at 26 °C (Fig. 4A) and 37 °C (Fig. 4B). The effect of active CpxR~P was consistently stronger on the relative transcription of *lpxP* compared to *lpxL*, and this was further accentuated by elevated temperature (Fig. 4). Importantly, the elevated expression in Δ*cpxA* was not observed in the complemented strain harbouring Δ*cpxA* that no longer accumulated active CpxR~P, whereas elevated expression remained in the mutant harbouring just the empty vector (Fig. 4). Further, compared to parental bacteria, active CpxR~P (in Δ*cpxA*) significantly reduced the relative expression of Lipid-A glycosylation genes, *arnB/pmrH/L-ara4N* (YPK_1831)*, arnF* (YPK_1837), *pmrE/ugd* (YPK_2077), *pmrC* (YPK_3740), and *pmrA/basR* (YPK_3741) (Fig. 4). Critically, this repression of gene expression was not observed in the complemented strain harbouring Δ*cpxA* that no longer accumulated active CpxR~P (Fig. 4). Hence, these data indicated a correlation between levels of accumulated active CpxR~P and regulation of targeted gene expression.

To confirm these observations, we utilised a set of isogenic strains based on the Δ*cpxR* mutant harbouring the empty expression vector pMMB208, or this vector with a clone of *cpxR* encoding for wild type CpxR (pCpxR_WT_ + ) or a clone of the *cpxR*_D8A, D9A, D51A, M53A, K100A_ mutant encoding for a CpxR variant lacking the active site for phosphorylation (pCpxR_Pneg_ + ). As elevated levels of CpxR are toxic to bacteria^35,54^ for this experimental setup we used the IPTG inducible promoter pMMB208 vector system instead of the native promoter pWKS30 vector system to permit tighter control of CpxR levels, and thereby avoid CpxR-dependent toxicity directed towards the host bacteria. Significantly, the Δ*cpxR/*pCpxR_WT_+ strain produced active CpxR~P, whereas the Δ*cpxR/*pCpxR_Pneg_+ only produced a non-phosphorylated CpxR inactive isoform, as judged by the Phos-tag^TM^ acrylamide gel system (Supplementary Fig. 1B). qRT-PCR analysis was performed using material derived from Δ*cpxR*/pMMB208, Δ*cpxR/*pCpxR_WT_+ and Δ*cpxR/*pCpxR_Pneg_ + . Once again, higher levels of active CpxR~P, this time in the Δ*cpxR/*pCpxR_WT_+ strain, resulted in elevated expression of both Lipid-A acylation genes, *lpxP* (YPK_430) and *lpxL* (YPK_1665) at 26 °C (Supplementary Fig. S6A) and 37 °C (Supplementary Fig. S6B) and reduced the relative expression of Lipid-A glycosylation genes, *arnB/pmrH/L-ara4N* (YPK_1831)*, arnF* (YPK_1837), *pmrE/ugd* (YPK_2077), *pmrC* (YPK_3740), and *pmrA/basR* (YPK_3741) at 26 °C (Supplementary Fig. S6A) and 37 °C (Supplementary Fig. S6B). On the other hand, production of the non-phosphorylated CpxR_Pneg_ inactive isoform by the Δ*cpxR/*pCpxR_Pneg_+ strain resulted in a reversal of these expression patterns at 26 °C (Supplementary Fig. S6A) and 37 °C (Supplementary Fig. S6B). This data collected from the Δ*cpxR* background mirrored the findings from the genetically independent Δ*cpxA* background. Thus, taken all together it provides compelling evidence for a crucial role of active phosphorylated CpxR~P in regulating positively acylation of Lipid-A, while concomitantly negatively regulating the glycosylation of Lipid-A.

### Direct binding of CpxR ~ P to the promoters of Lipid-A acylation and glycosylation genes

**Fig. 5:**
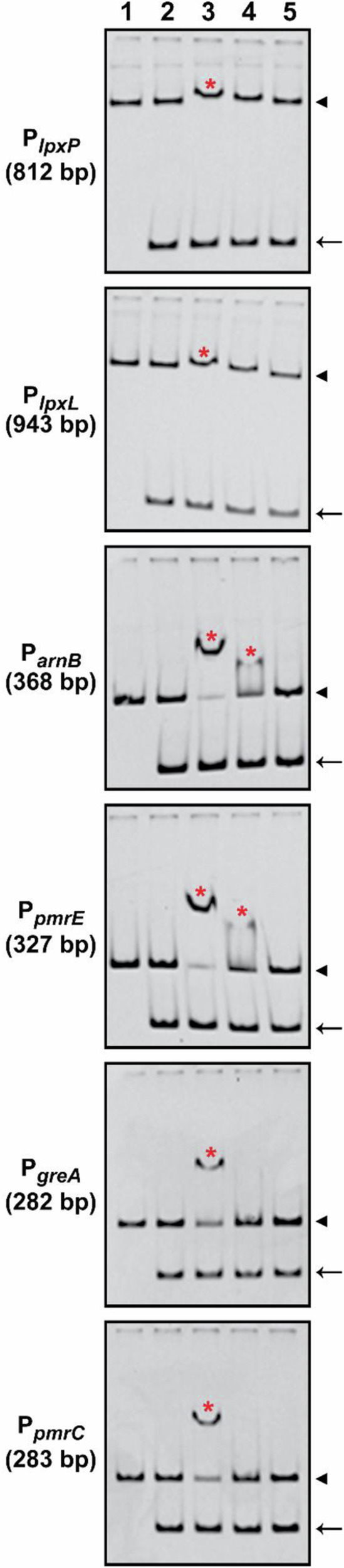
Active phosphorylated CpxR~P binds to the promoters of Lipid-A acylation and glycosylation genes. EMSAs with the complete 5’ intergenic regulatory DNA regions of indicated Lipid-A remodelling gene targets were mixed with active CpxR~P to measure specific protein-nucleic acid interactions. Red asterisks (*) indicate the target promoter DNA-CpxR_WT_ complex. Unbound promoter DNA is indicated with an arrowhead (◄). The inactive non-phosphorylatable CpxR_Pneg_ isoform was unable to bind target DNA under the same conditions. A 16S rDNA fragment (148 bp) used as a ‘non-specific’ negative control and its running location is indicated by an arrow (←). Lane-1: target promoter DNA, Lane-2: target promoter plus non-specific 16S rDNA, Lane-3: target promoter, non-specific 16S rDNA and CpxR_His6_ (100 µM), Lane-4: target promoter, non-specific 16S rDNA and CpxR_His6_ (50 µM) and Lane-5: target promoter, non-specific 16S rDNA and phosphorylation defective CpxR_Pneg-His6_ (100 µM). Each reaction contained 200 mM Acetyl phosphate to phosphorylate CpxR_His6_ in the respective lane. Gels were stained with 1× GelRed DNA-staining dye solution.

Having established that active phosphorylated CpxR~P stimulates the transcription of lpxP and lpxL involved in Lipid-A acylation and represses the transcription of *arnB/pmrH/L-ara4N*, *arnF*, *pmrE/ugd*, *pmrC* and *pmrA/basR* involved in Lipid-A glycosylation, we questioned if this regulatory control occurred via direct promoter binding by the response regulator. We first PCR amplified promoter regions of the acylation operon genes, lpxP (P_YPK_0430_) and lpxL (P_YPK_1665_) as described in Supplementary Fig. S4, and the promoter regions of the glycosylation operon genes, *arnB/pmrH/L-ara4N* (P_YPK_1831_), *pmrE/ugd* (P_YPK_2077_), *pmrA/basR* (P_YPK_3739_) and *pmrC* (P_YPK_3740_) as defined in Supplementary Fig. S5. To examine binding of purified wild-type CpxR_His6_ to these promoters the concentrations of 50 µM and 100 µM were used in gel mobility shift assays. In all cases, the higher concentration of active phosphorylated CpxR_WT_ caused a shift of all targeted promoter DNA fragments (Fig. 5). This was specific targeted binding because inactive non-phosphorylated CpxR_Pneg_ failed to induce any shift on these promoter DNA fragments under identical conditions, and neither CpxR_WT_ nor CpxR_Pneg_ induced a shift of the 16S rRNA encoding DNA fragment that was used as another specificity control (Fig. 5). Hence, phosphorylated CpxR~P binds to the promoters of the Lipid-A acylation and glycosylation modifying genes, most probably at sites that containing a near consensus CpxR binding box (Supplementary Fig. S4 and Fig. S5). Thus, we attribute the enhanced susceptibility to colistin and polymyxin B in Δ*cpxA* to enhanced CpxR~P that drives expression of lpxP and lpxL through direct promoter binding and which alters the balance of Lipid-A acylation and Lipid-A glycosylation. This direct binding is likely the key transcriptional regulatory mechanism driving CpxR~P dependent controlled modulation of Lipid-A species that influences outer membrane integrity and antibiotic susceptibility.

## Discussion

**Fig. 6:**
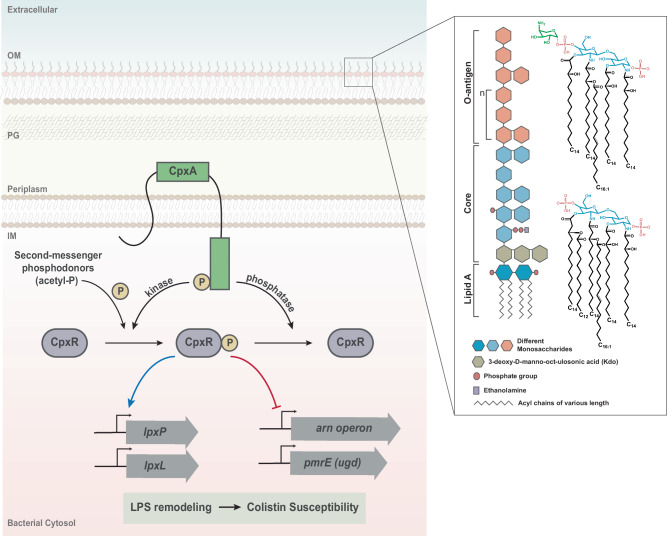
A working model of CpxR~P mediated Lipid-A remodelling in *Y. pseudotuberculosis*. Cellular location of the CpxAR two-component system is depicted with its functionality on the Lipid-A remodelling and colistin susceptibility. A zoomed in version of the outer membrane (OM) constituent, Lipopolysaccharide (LPS) is illustrated with individual components. CpxR~P effect on promoter of the LPS remodelling genes and/or operons is represented either with blue arrow (+ or positive regulation) or a red hammer headline (− or negative regulation).

In this study, we established that the CpxA-CpxR TCST is important for outer membrane remodelling in *Yptb* through modification of the Lipid-A core of LPS, and we present our working model of this mechanism in Fig. 6. A consequence of this remodelling is altered susceptibility to colistin and polymyxin B. In bacteria that accumulate high levels of the active CpxR~P isoform, such as the Δ*cpxA* mutant used in this study, outer membrane remodelling is pronounced and colistin and polymyxin B susceptibility is clear. The influence of accumulated active CpxR~P isoform involves direct promoter binding and transcriptional activation of lpxP and lpxL involved in Lipid-A acylation and transcriptional repression of *arnB/pmrH/L-ara4N*, *arnF*, *pmrE/ugd*, *pmrC* and *pmrA/basR* involved in Lipid-A glycosylation. This regulatory connection occurs regardless of growth temperature. This suggests any link between the Cpx pathway and temperature is not merely due to temperature imposing stress on membranes. These findings extend our understanding of the important roles played by Cpx-signalling in maintaining the adaptability and pathophysiology of the clinically relevant *Yptb* model bacterium when exposed to extracytoplasmic stress.

Multiple molecular mechanisms conferring colistin activity and resistance are well established^5^. The action of cationic colistin or polymyxin B involves electrostatic interactions with anionic Lipid-A molecules leading to permeability changes in the cell envelope and consequently bacterial killing^5^. It follows that the most common resistance mechanisms concern Lipid-A modification by the addition of cationic groups phosphoethanolamine (pEtN) or 4-amino-L-arabinose (L-Ara4N)^5^. Elevating Lipid-A cationisation diminishes electrostatic interactions with colistin conferring resistance, which is true for parental *Yptb*. However, accumulated active CpxR~P isoform reduced L-Ara4N modification of Lipid-A by direct transcriptional repression of the *arn* and *pmr* operons. In these bacteria, predominately unmodified Lipid-A remains negatively charged, which enhances interactions with cationic colistin conferring susceptibility.

Interestingly, we also identified CpxR-dependent transcriptional activation of the *lpxP* and *lpxL* genes, potentially leading to changes in Lipid-A acylation (Fig. 5). Despite that, our MALDI data did not show a pronounced effect in vivo, as hexa-acylated Lipid-A remained detectable in all conditions (Fig. 3). This suggests the presence of additional mechanisms that control hexa-acylation of Lipid-A. Nevertheless, any change to the acylation pattern of Lipid-A - even if not leading to a complete abrogation of hexa-acylated Lipid-A biosynthesis - might impact outer membrane barrier function through changes in the physical, mechanical, and chemical parameters of the highly asymmetric lipid bilayer and their effects on outer membrane protein biogenesis^55^. Moreover, we were unable to observe in our MALDI data alterations in PmrC-dependent phosphoethanolamine modification of Lipid-A. This is despite observing that CpxR~P binds to the *pmrC* promoter (Fig. 5) and that CpxR~P accumulation repressed *prmC* gene expression (Fig. 4). This suggests that our current qualitative approach lacked the sensitivity to detect PmrC-dependent alterations. We did attempt quantitative TLC-based methods such as published extensively by the Trent laboratory^56^ but obtained fractions of Lipid-A fell short of detectability thresholds.

These data suggest that Cpx-signalling activation by exposure to extracytoplasmic stresses is a trade-off in *Yptb* fitness. Active CpxR~P controls the expression of those genes whose products can restore protein quality control in the bacterial envelope that is essential for maintenance of membrane integrity^19,20^. However, this process has collateral damage in the form of CpxR~P control of Lipid-A modification that renders *Yptb* susceptible to killing by cationic antibiotics, such as colistin and polymyxin B. In part, this collateral damage would be marginalised because the CpxAR system is tightly feedback inhibited to avoid activation under inappropriate conditions or excessive build-up of active CpxR~P that can be toxic for cells^54,57–63^. However, since bacteria are masters of adaptability to achieve optimal fitness in their prevailing environment, collateral damage in a subpopulation may be tolerated to achieve fitness among most of the cells within the population. Awareness of phenotypic heterogeneity within a bacterial population during the process of adaptation is becoming increasingly common. The concept is viewed in terms of “bet-hedging”, which suggests that a cell population with diverse heterogeneous phenotypic traits can as a collective out-perform cell populations exhibiting homogeneous phenotypic traits^64–66^. Hence, a future goal would be to utilise recently developed single-cell technologies^67–70^ to elucidate the stochastic CpxR-dependent regulatory events responsible for “bet-hedging” phenotypic heterogeneity in single cells within populations of *Yptb*-YPIII.

Independent whole transcriptomic studies have revealed an extensive CpxR-regulon in several bacteria^71–74^. Hence, the extended network of CpxR control means that the targeting of the genes encoding for glycosylation and acylation pathways within Lipid-A biosynthesis may not be the sole reason for *Yptb-*YPIII sensitivity to colistin and polymyxin B. Indeed, Lipid-A modification is not the only aspect affecting the membrane permeability barrier. Several CpxR-regulated genes encode envelope proteins associated with membrane integrity and function^45–47^. Most envelope proteins function as assembly platforms or are involved in solute or protein translocation, as well as signal transduction and cell division. In which case, they are localised to potentially influence the influx or efflux of antibiotics. Importantly, recent reports revealed that CpxR-mediated control of envelope efflux transporters in diverse Gram-negative bacteria correlated with resistance profiles to various antibiotics and antimicrobial peptides^75–78^. Moreover, the Cpx-signalling system has been linked to regulation of porin levels in *E. coli*^21,45,79,80^, and in other bacteria^81–84^. This is relevant since a systemic mutational study in *E. coli* demonstrated discrete roles for OmpA and OmpC porins in membrane integrity, whereas the OmpC and OmpF porins contribute to antibiotic resistance^15^. Hence, it is worth exploring potential connections between Cpx-signalling and solute transport activity in *Yptb-*YPIII as a further means to establish mechanisms contributing to membrane permeability and drug susceptibility.

This study sought to identify the molecular mechanism for the effects of Cpx-signalling on Lipid-A modifications. Several molecular and biochemical experiments detailed in this study identified a direct effect of accumulated active CpxR~P isomer on the expression of Lipid-A modification operons. It is likely that this mechanism predominates under certain extracytoplasmic stresses. However, other players may contribute more under alternate conditions. Indeed, it is established in other bacterial model systems that signalling through the two component systems PmrA/PmrB, PhoP/PhoQ and possibly even ArcA/ArcB influence Lipid-A modification^5,85–87^. Naturally, we aim in follow-up studies to investigate the existence of overlapping regulatory mechanisms between two or more TCST systems in *Y. pseudotuberculosis*. There is already precedent for genetic interactions between the Cpx-signalling system and the Arc system^88,89^, the Pho system^89^, and the Pmr system^90^ in other bacteria, but the nature of this overlap is not always clear.

We report herein the susceptibility of *Yptb* to polycationic antibiotic colistin and polymyxin B. However, observations from others have shown that alterations in Cpx-signalling modulate bacterial sensitivity to a range of hydrophobic antimicrobials and antibiotics^38,81,91^. Nevertheless, the sensitivity caused by accumulation of CpxR~P is not observed for all antibiotic classes^91^. Hence, understanding the contributions of Cpx-mediated outer membrane remodelling and antimicrobial sensitivity phenotypes could reveal unique mechanisms of bacterial killing for certain antibiotics. This is the incentive to investigate the responsiveness of the *Yptb* CpxA-CpxR TCST system to a range of antibiotic and antimicrobial peptide exposures, and to determine the capacity for accumulated CpxR~P to subsequently alter *Yptb* sensitivity to these antimicrobials. Importantly, *Yptb* is an excellent clinically relevant model system for these investigations, as evidenced by this study that defined a molecular link between Cpx-signalling and control of Lipid-A remodelling and colistin susceptibility. Nevertheless, since variations within the molecular makeup of the cell envelope exists among different bacteria^55,92–97^, knowledge collected from a range of *Yersinia* clinical isolates as well as other unrelated bacterial pathogens concerning the role played by the Cpx response in regulating virulence and antibiotic resistance will help give impetus to finding novel antimicrobials and understand their molecular targets in the cell envelope.

As membrane permeability and drug susceptibility are interwoven, opportunities to remodel the outer membrane provides possibilities for novel antibiotic development and thereby reverse certain antibiotic resistance phenotypes^98^. Given that Cpx-signalling is linked to the control of these two vital physiological properties, the Cpx pathway is an attractive target for much needed new antimicrobial development. This is made more relevant because the Cpx pathway is highly conserved in Gram-negative bacteria^27,74,84^, and it plays a role in virulence gene expression control in multiple diverse clinically important bacteria^23–31^. Indeed, chemicals that interfere with Cpx-signalling have been identified that modulate Cpx-dependent virulence phenotypes in clinically relevant bacteria^74,99–101^. Thus, the Cpx pathway could be a new broad-spectrum target for developing new drugs for overcoming bacterial resistance to conventional antibiotic resistance.

## Methods

### Bacterial strains and growth conditions

Bacterial strains used in this study are listed in supplementary Table S1. The parental strain (designated wild type – WT) *Y. pseudotuberculosis* YPIII/pIB102 (serotype III) is an original clinical isolate^102^ and harbours the pIB102 virulence plasmid encoding for the Ysc-Yop T3SS. It also encodes a disruptive kanamycin resistance cassette inserted into the *yadA* gene^103^, and a defective *phoP* allele that yields a non-functional PhoP/Q regulatory cascade^43^. All mutants used in this study are isogenic to the parental strain. YPIII07/pIB102 strain encodes a Δ*cpxA* in frame deletion of nucleotides encoding codons 41 to 449, and the isogenic YPIII08/pIB102 strain encodes a Δ*cpxR* in frame deletion of nucleotides encoding codons 11 to 193. Complementing plasmids capable of ectopic expression of *cpxA* or *cpxR* alleles are described in supplementary Table S1. Bacteria were routinely cultivated in either lysogenic broth (LB) or Mueller-Hinton broth (MHB) with aeration or agar at 26 °C or 37 °C. When required, antibiotics were added at a final concentration of kanamycin sulphate (11815-024) (50 µg/ml), colistin sulfate salt (C4461) (8, 4, 2, 1, 0.5, 0.25, 0.125, 0.625 and 0.3125 µg/ml) or polymyxin B sulfate (PHR1595) (8, 4, 2, 1, 0.5, 0.25, 0.125, 0.625 and 0.3125 µg/ml). Growth curves where performed in Falcon 96-well plates and OD600 was monitored for 40 h in a Fluostar Omega plate reader (BMG Labtech) for each time point.

### Antibiotic susceptibility testing and time-kill assays

Colistin and polymyxin B susceptibility testing involved determination of the MICs by broth microdilution according to the guidelines of the CLSI/EUCAST and performed essentially, as described previously^104^. Briefly, overnight culture of parental strain (WT), Δ*cpxA*, Δ*cpxA*/pWKS_*cpxA* and Δ*cpxA*/pWKS were grown in MHB overnight in 50 ml flasks. Cultures were diluted and used at a final inoculum of 5 × 10^6^ CFU per well. Cell star (655 180) 96-well plates containing the appropriate dilution of antibiotic (4 μg/ml to 0.125 μg/ml) or growth control were inoculated. Plates were checked after 20 h of growth at 26 °C and the lowest concentration of colistin or polymyxin B that inhibits the visible growth was determined. Quantification of inoculum was performed for each assay using serial dilutions and plating.

For the antibiotic time-kill assays, the plates were set essentially as described above but left for 1 h at 26 °C with the colistin or polymyxin B range of (4 μg/ml to 0.125 μg/ml) and antibiotic negative growth controls. Samples were then serially diluted in MHB in Falcon (351172) 96 well plates and 20 µl spotted onto LB plates and grown at 26°C for quantification of CFUs.

### Negative stain electron microscopy imaging

To assay for the effect of colistin on the bacterial cell envelope, overnight cultures of the parental strain (WT) and Δ*cpxA* mutant as well as the complementing strain Δ*cpxA/*pWKS_*cpxA* and an empty plasmid control Δ*cpxA/*pWKS were inoculated in fresh MHB and grown at 26°C until an OD_600_ of 1.0. Cultures were then supplemented with 2 µg/ml of colistin and incubated for a further 30 min. This concentration of colistin corresponds to four-times the calculated MIC of the parental (WT) strain to exert a pronounced effect on the cell envelope without prolonged incubation times. A volume of 10 µl of bacterial cell cultures was then deposited over glow discharged 300 mesh carbon-coated grids for 2 min followed by two washing steps before staining for 10 s with 0.04% w/v of phosphotungstic acid (PTA). Images were later recorded on a Tecnai Spirit electron microscope operating at 120 kV.

### Isolation of Lipid-A

Overnight cultures were inoculated into 7 ml of fresh LB and incubated at 26 °C or 37 °C until the mid-exponential phase (equivalent to an OD_600_ between 0.6 to 0.8). Cultures were pelleted, resuspended in 1 ml of LB, and heat-inactivated for 1 h at 80 °C. Bacteria were then washed five times with distilled water to remove any residual salt contamination that could affect ionization during mass spectrometry analysis. Subsequently, acid hydrolysis was performed for all samples to induce carbohydrate hydrolysis from the Lipid-A moiety^105^, thereby improving Lipid-A binding to the MALDI matrix and signal-to-noise ratio of the final spectra. Samples were treated with 1% acetic acid (v/v) for 2 h at 100 °C followed by centrifugation at 17000 × g for 15 min. Supernatants were discarded and pellets containing hydrolysed cells were resuspended in 100 µl of distilled water.

### MALDI analysis

Aliquots of 0.4 µl for each acid-treated sample were loaded onto a 384-well MALDI plate and immediately overlaid with 0.8 µl of a super-2, 5-dihydroxybenzoic acid matrix prepared at a final concentration of 10 mg/ml in chloroform/methanol (9:1, v/v). Bacterial samples and matrix were mixed by pipetting and left to air dry. MALDI-TOF MS analysis was performed on a 4800 Proteomics Analyzer (Applied Biosystems, Foster City, CA, USA) using the reflectron mode. A voltage of 20 kV in the negative ion mode was applied using an extraction delay time set at 20 ns and allowed analysis of Lipid-A species in each sample. All MS datasets were analysed using the Data Explorer software (Applied Biosystems, version 4.9).

### RNA isolation and transcriptional analysis by quantitative real-time PCR

The detailed procedure is available elsewhere^30^. In brief, bacterial cultures were equalised to the lowest obtained OD_600_ to ensure the same cell density, then total RNA was isolated using the NucleoSpin RNA isolation kit (Macherey-Nagal, Germany). Isolated total RNA was treated with Turbo DNase and inactivated as recommended using DNase inactivation reagent. This RNA was used to synthesise cDNA using RevertAid H-minus reverse transcriptase (ThermoFisher). A negative reaction (without reverse transcriptase) was also prepared and used as a DNA contamination check. Synthesised cDNA was quantified by a Nano-drop spectrophotometer and stored at -20 °C. Real-time qPCR was performed from 100 ng of cDNA template and the gene-specific qRT-primer pairs listed in supplementary Table S2. Relative expression of each gene was calculated by the Livak method (2^-∆∆Ct)^106^ and converted into Log_2_-fold change to examine the impact of CpxR~P as an activator or repressor for the gene of interest. An internal expression (without normalisation with internal standards) also checked to assess the internal variation among each sample.

### In vitro binding of active and phosphorylation defective CpxR variants

The electrophoretic mobility shift assay (EMSA) was used to test in vitro binding of the purified wild-type (active) CpxR_WT_ and phosphorylation defective CpxR_Pneg_ (CpxR_D8A_, _D9A_, _D51A_, _M53A_, _K100A_) His_6_ variants to the predicted regulatory region of *lpxP* (P_YPK_0430_), *lpxL* (P_YPK_1665_), *arnB/pmrH/L-ara4N* (P_YPK_1831_), *pmrE/ugd* (P_YPK_2077_), *pmrA/basR* (P_YPK_3739_) and *pmrC* (P_YPK_3740_). The regulatory regions of these gene targets, encompassing potential CpxR-binding site(s) predictions are depicted in supplementary Fig. S4 and supplementary Fig. S5. Gene-specific PCR amplification used the EMSA primer pairs listed in Table S2. Expression and purification of both wild-type CpxR_WT_ and phosphorylation defective CpxR_Pneg_ variants and EMSA reactions were performed as described previously^30^.

### Phos-tag^TM^ of in vivo accumulated CpxR ~ P

Mass spectroscopy was previously used to pinpoint the phosphorylated amino acid in in vitro purified CpxR^32^. To visualise in vivo phosphorylated CpxR we used the Phos-tag^TM^ technology. The procedure followed is detailed elsewhere^30^. Often two or more bands appear for the CpxR~P product. We assume this is due to the chemistry of the Phos-tag acrylamide gel and its affinity for the phosphorylated CpxR~P, and due to the inherent instability of the phosphorylated isomers. These issues have been documented elsewhere^107,108^.

### Statistical analysis

Statistical analysis was performed using GraphPad Prism version 10. Overnight OD_600_ and CFU per ml for the parental strain (WT) and *cpxA* mutant were compared using a T-test. For analysis of the CFUs and ODs of the colistin survival assays over time Two-way ANOVA with Tukey’s multiple comparisons test was performed. One-way ANOVA with Tukey’s multiple comparisons test was performed to compare relative CpxR~P levels.

## Supplementary information

Supplementary information

## Data Availability

The datasets generated and/or analysed during the current study are available from the corresponding authors upon reasonable request.
