# Supplementary material for: Cpx-signalling in Yersinia pseudotuberculosis modulates Lipid-A remodelling and resistance to last-resort antimicrobials
Source: NPJ Antimicrob Resist. 2024 Nov 18;2:39. doi: 10.1038/s44259-024-00059-y (PMC11573712; doi:10.1038/s44259-024-00059-y)
Supplement: Supplementary file 1 — Supplementary information [file 44259_2024_59_MOESM1_ESM.pdf]

Cpx-Signalling in *Yersinia pseudotuberculosis* Modulates Lipid-A Remodelling and Susceptibility to Last-Resort Antimicrobials

Dharmender K. Gahlot, Jonasz B. Patkowski, Jaime Fernández de Santaella, Luke P. Allsopp, Zhiqiao Pan, Alain Filloux, Gerald Larrouy-Maumus, Matthew S. Francis, Tiago R. D. Costa

Supplementary material

Table S1. Bacterial strains and plasmids used in this study.

Table S2. Oligonucleotides used in this study

Supplementary Figure S1. *In vivo* accumulated CpxR~P in *Y. pseudotuberculosis*.

Supplementary Figure S2. Growth curve of *Y. pseudotuberculosis* isogenic mutants and complemented strains.

Supplementary Figure S3. Loss of CpxA renders *Y. pseudotuberculosis* more susceptible to polymyxin B.

Supplementary Figure S4. Organisation of *lpxE* and *lpxL* genetic environments.

Supplementary Figure S5. Organisation of *arnB, pmrE* and *pmrC* genetic environments.

Supplementary Figure S6. Gene specific transcription is altered by modulation of CpxR levels in *Y. pseudotuberculosis.*

Table S1: Bacterial strains and plasmids used in this study.

Strain or plasmid	Relevant genotype or phenotype[1]	Source or reference
***Y. pseudotuberculosis* strains**		
YPIII/pIB102 (parent)	pIB102, *yadA*::Tn5, PhoP$^-$, Km^R	Wolf-Watz, et al. [1]
YPIII07/pIB102 (Δ*cpxA*)	pIB102, *yadA*::Tn5, PhoP$^-$, *cpxA* in frame deletion of codons 41 to 449, Km^R	Carlsson, et al. [2]
YPIII08/pIB102 (Δ*cpxR*)	pIB102, *yadA*::Tn5, PhoP$^-$, *cpxR* in frame deletion of codons 11 to 139, Km^R	Carlsson, et al. [2]
**Plasmids**		
pWKS30	Low copy number cloning plasmid, Amp^R	Wang and Kushner [4]
pJF067 ($\mathrm{pCpxA}_{\mathrm{wt}}$)	1549 bp synthetic DNA fragment of *cpxA* (full length) with its native promoter cloned into XbaI-XhoI digested pWKS30, Amp^R	Thanikkal, et al. [5]
pMMB208	Expression plasmid, Cml^R	Morales, et al. [6]
pKEC021	747 bp XbaI/KpnI PCR fragment of *cpxR* in pMMB208, Cml^R	Carlsson, et al. [2]
pDK1007 ($\mathrm{pCpxR}_{\mathrm{Pneg}}$)	747 bp XbaI/KpnI PCR fragment of $cpxR_{\mathrm{D8A,\ D9A,\ D51A,\ M53A,\ K100A}}$ in pMMB208, Cml^R	Gahlot, et al. [7]
pET22b(+)	Expression vector, Amp^R	Novagen
pKEC017	~700 bp NdeI/XhoI PCR fragment of *cpxR* in pET22b(+) that creates a $\mathrm{His}_{(6)}$ C-terminal fusion, Amp^R	Carlsson, et al. [2]

[1]Cml^R: Chloramphenicol-resistant, Km^R: Kanamycin-resistant, Amp^R: Ampicillin-resistant

Table S2. Oligonucleotides used in this study.

Primer pair	Sequence (5' to 3')	Target	Amplicon size (bp)
qRT-PCR analysis			
1831-qRTf and 1831-qRTr2	CAAAGCCATTATTCCCGTCC and CCAATCCATTGATCTCGATAAC	*arnB*	141
1837-qRTf and 1837-qRTr2	GCAAGCGTCGTATTGGTCAC and CATCAAACCACACATTACTGATGC	*arnF*	144
3741-qRTf and 3741-qRTr	ACCAGTCAGTACAGCATGATTATTC and CCTGTCTTCAAGCGCATCAC	*pmrA/basR*	138
3740-qRTf and 3740-qRTr2	TCAGGTCGAGAATTACACTCAG and CCATCTGCTGAGCGTGATAG	*pmrC*	110
2077-qRTf and 2077-qRTr2	GCTGCCGATCTTAAATATGTCC and ACTTGATGTACTTTATCCGCTG	*pmrE*	122
1665-qRTf and 1665-qRTr	TCTATGCGATGGGAACATCACTG and ACGTTCCTCTGGTGACATATGG	*lpxL*	116
0430-qRTf and 0430-qRTr	TACCCACTACTCAATAAGTTAGGTG and CAAGGACTTGTTTATCCATGTCAG	*lpxP*	127
qRT-16SrRNAF4 and qRT-16SrRNAR4	TTTGTTGCCAGCACGTAATGGT and GCGAGTTCGCTTCACTTTGTATCT	16S rRNA	147
0004-qRTf and 0004-qRTr	GGACAACGCTATTGACGAAGC and CCTTCCTCATCGTGCATACC	*gyrB*	129
0341-qRTf and 0341-qRTr	CTTCTCCAACTGCGCACATC and GACGTTCCAGGTTGGTCATAC	*rpoC*	147
EMSA analysis			
EMSA-0430F and EMSA-0430R	CATAGCAATACCTTCTTG and CATATGAACTCTATCGTTATGC	Lipid A biosynthesis lauroyl (or palmitoleoyl) acyltransferase (*lpxP*)	812
EMSA-1665F and EMSA-1665R	CTGCATTGTCCAGCCTCTG and CATTATCAGTTCTCAGCGTC	Lipid A biosynthesis lauroyl (or palmitoleoyl) acyltransferase (*lpxL*)	943
EMSA-1831F and EMSA-1831R	CACAAGAAGCGTATAACGCTTAG and CATTAATAAATCCCTTAGGACAC	*L-ara4N*- Amino sugar and nucleotide sugar metabolism pathway-Involved in Phosphorelay system, transfer of PO4^{-} transfer	368
EMSA-2077F and EMSA-2077R	CTTGCTGCTGAAGAATAGTCATC and CATTCGCTCGATCTCCGAGTTG	UDPglucose 6-dehydrogenase (*pmrE*)	327
EMSA-3739F and EMSA-3739R	GCATAATTTCGGTATAACTGC and CGTCATCGGAATCTGTTTCATC	Transcription elongation factor GreA; Promoter for PmrA/BasR (*greA*)	282
EMSA-3740F and EMSA-3740R	GTCATCGGAATCTGTTTCATC and ATGCATAATTTCGGTATAACTGC	Serine-type D-Ala-D-Ala carboxypeptidase/endopeptidase OR Penicillin-binding protein 4 (*pmrC*)	283

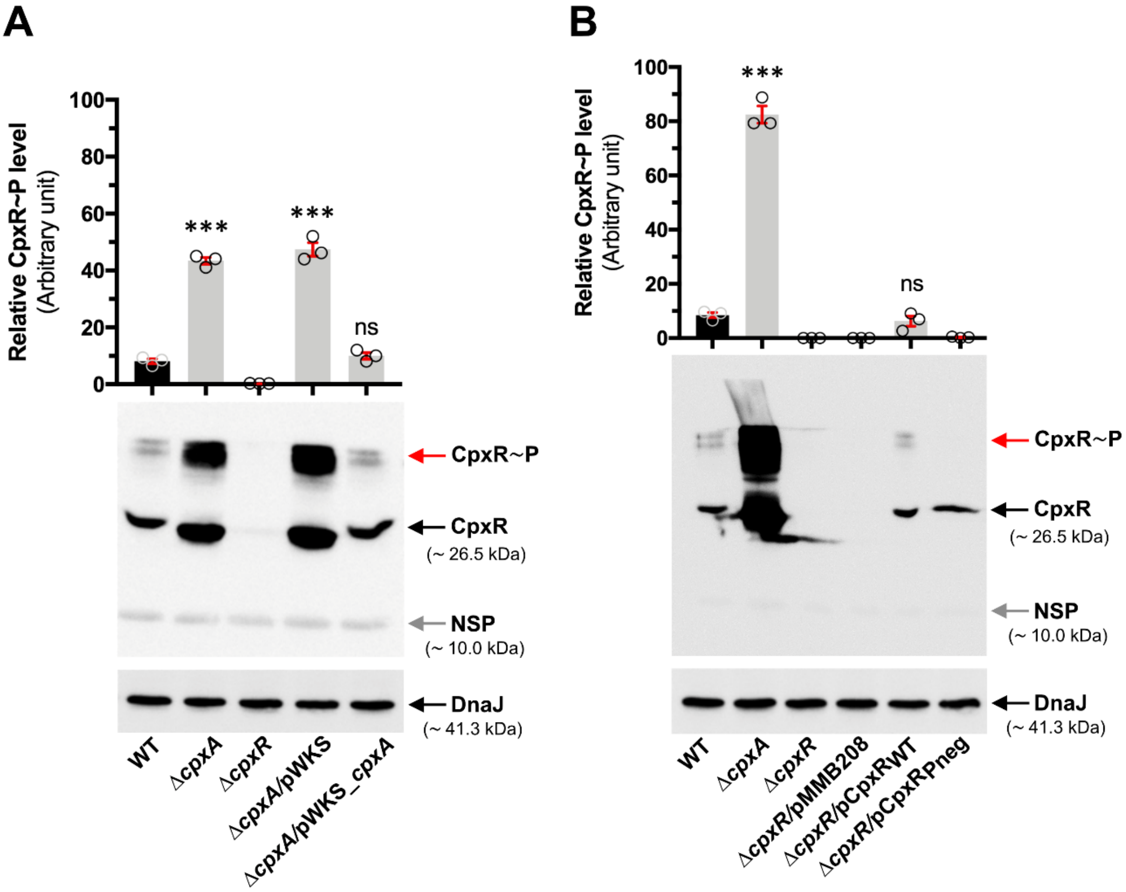

Supplementary Figure S1. *In vivo* accumulated CpxR~P in *Y. pseudotuberculosis*.
The Phos-tag^{TM} acrylamide system [8,5,7] was used to measure accumulated CpxR~P in bacteria grown in LB broth to late stationary phase at 26°C with aeration. Lysed bacteria were electrophoresed on a freshly prepared 12% Phos-tag^{TM} Acrylamide AAL-107 gel, immunoblotted, and detected with anti-CpxR antiserum. Assay specificity was validated using the $\text{CpxR}_{\text{Pneg}}$ mutant variant that separated as only the non-phosphorylated isomer. For this variant, a phosphorylated form was not detectable, even after prolonged exposure of the immunoblot. Antibody specificity was controlled by inclusion of the Δ*cpxR* null mutant that lacked any detectable production of CpxR. The cytoplasmic molecular chaperone DnaJ served as a loading control. Strains: parent (WT); Δ*cpxA* null-mutant; Δ*cpxR* null mutant; Δ*cpxA* null mutant/pWKS_*cpxA*; Δ*cpxA* null mutant/pWKS (empty vector); Δ*cpxR* null mutant/pMMB (empty vector); Δ*cpxR* null mutant/pCpxR_{WT}; Δ*cpxR* null mutant/$\text{pCpxR}_{\text{Pneg}}$. The red arrow reflects the active phosphorylated CpxR isoform accumulated in the *Yersinia* cytoplasm, while the black arrow indicates the accumulated inactive non-phosphorylated CpxR isoform. An

unknown degradation product (NSP; non-specific protein) is indicated by a grey arrow. A statistical significance between accumulated CpxR~P levels from the various strains with respect to the parent (WT) was determined using One-way ANOVA with Tukey's multiple comparisons test, with a single pooled variance. The difference in variance with a *p*-value of <0.05 was considered significant. The *p*-values are indicated by <0.001 (***) and >0.05 (ns; non-significant). Quantitative data is derived from three biological replicates and the panels are representative of one of these replicates.

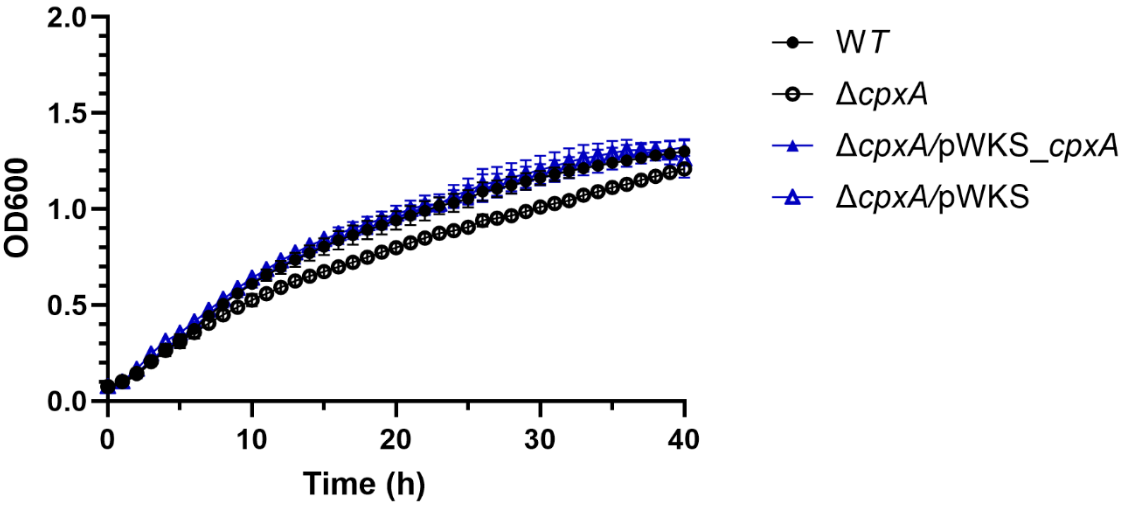

Supplementary Figure S2. Growth curves of *Y. pseudotuberculosis* isogenic mutants and complemented strains.

Growth was measured at 26°C in MHB for 40 h, N = 3 independent replicates. Parental strain (WT); Δ*cpxA* null-mutant; Δ*cpxA* null mutant/pWKS_*cpxA*; and Δ*cpxA* null mutant/pWKS (empty vector).

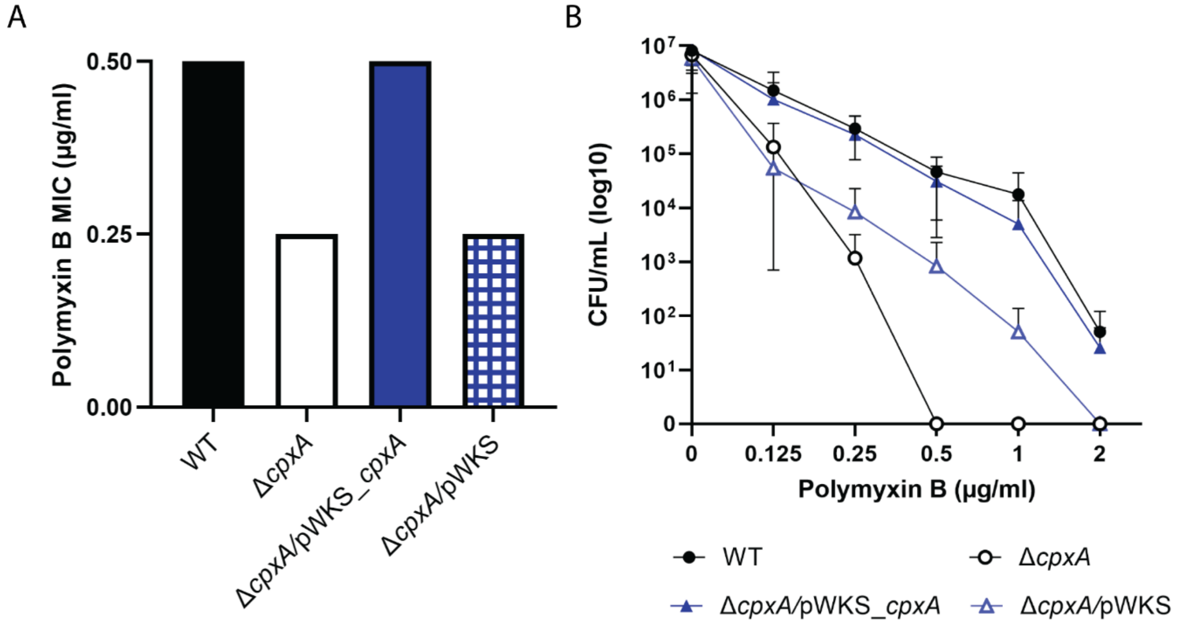

Supplementary Figure S3. Loss of CpxA renders *Y. pseudotuberculosis* more susceptible to polymyxin B.

(A) Minimal inhibitory concentrations to polymyxin B of our parent strain (WT)**,** Δ*cpxA***,** Δ*cpxA/*pWKS_*cpxA***,** Δ*cpxA*/pWKS strains. Representative of at least three separate experiments. **(B)** 1-hour polymyxin B time-kill assay plotted as colony forming units shows enhanced killing of Δ*cpxA* and Δ*cpxA*/pWKS strains compared to parent or Δ*cpxA/*pWKS_*cpxA*. The difference between parent or complemented (Δ*cpxA/*pWKS_*cpxA*) against Δ*cpxA* or Δ*cpxA/*pWKS is statistically significant (for example concentration 0.25, $P<0.05$, Two-way ANOVA Tukey's multiple comparison test). The graph depicts data from three biological experiments.

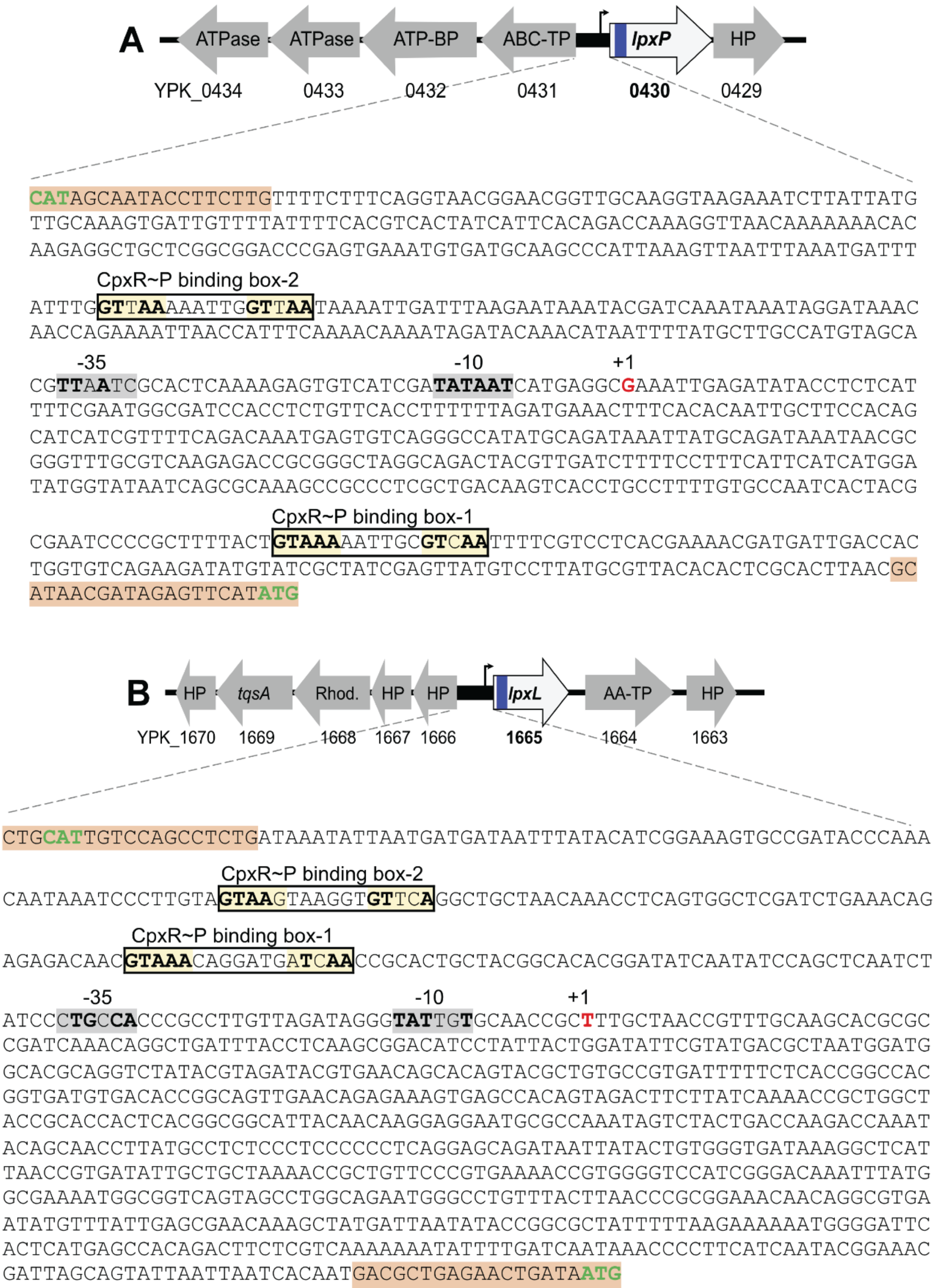

Supplementary Figure S4. Organisation of *lpxE* and *lpxL* genetic environments.

The locus-tag (YPK_XXXX) of each gene is mentioned underneath. PCR-amplified gene-specific (for qRT-PCR) and 5’ UTR (for EMSA) fragments are represented by respective vertical blue and horizontal black rectangle box. The promoter regions P_{lpxP} (A) and P_{lpxL} (B) upstream of *lpxP* and *lpxL* of *Yptb*-YPIII is the zoomed-out DNA sequence delineated by the

positions of the forward and reverse primers (in orange highlight). These sequences were used as the EMSA template described in Figure 4, and shows potential CpxR~P binding motifs that would present on the sense strand (yellow highlight) that are very similar to the established CpxR~P DNA-binding consensus sequence, 5'-GTAAA$(N)_{4-8}$ GTAAA-3'[9,10]. Potential Sigma-70 based promoter elements, −35 and −10, are bold and highlighted in grey. The +1 nucleotide in red identifies a potential transcriptional start site and the codon in green is that translational start site.

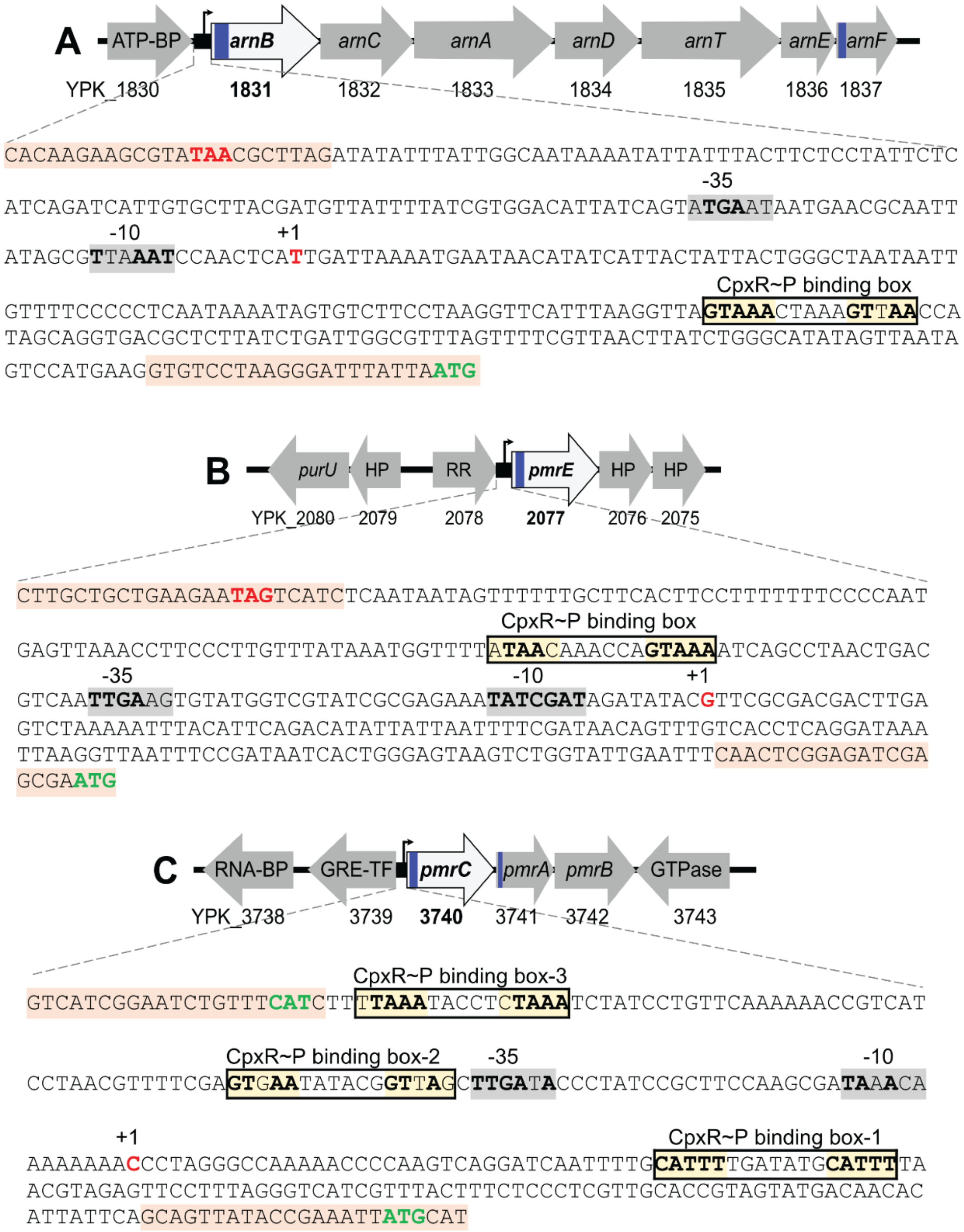

Supplementary Figure S5. Organisation of *arnB, pmrE* and *pmrC* genetic environments. The locus-tag (YPK_XXXX) of each gene is mentioned underneath. PCR-amplified gene-specific (for qRT-PCR) and 5’ UTR (for EMSA) fragments are represented by respective vertical blue and horizontal black rectangle box. The promoter regions P_{arnB} (A), P_{pmrE} (B) and P_{pmrC} (C) upstream of *arnB, pmrE* and *pmrC* of *Yptb*-YPIII is the zoomed-out DNA sequence delineated by the positions of the forward and reverse primers (in orange highlight). These sequences were used as the EMSA template described in Figure 4, and shows potential CpxR~P

binding motifs that would present on the sense strand (yellow highlight) that are very similar to the established CpxR~P DNA-binding consensus sequence, 5’-GTAAA(N)$_{4-8}$ GTAAA-3’[9,10]. Potential Sigma-70 based promoter elements, −35 and −10, are bold and highlighted in grey. The +1 nucleotide in red identifies a potential transcriptional start site and the codon in green is that translational start site.

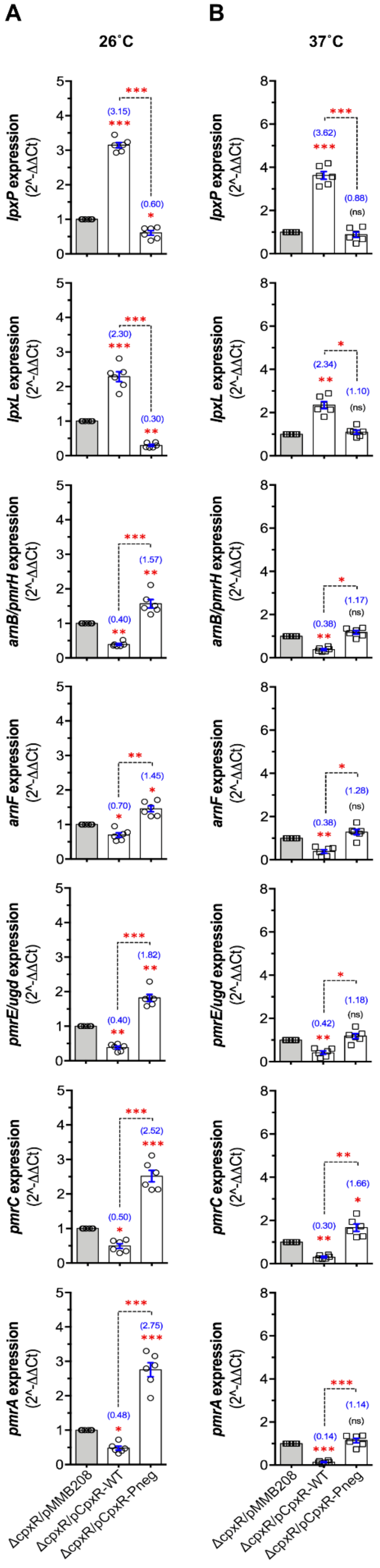
A
B
26˚C
37˚C
lpxP expression (2^-ΔΔCt)
(3.15)
(0.60)
(3.62)
(0.88)
(ns)
lpxL expression (2^-ΔΔCt)
(2.30)
(0.30)
(2.34)
(1.10)
(ns)
arnB/pmrH expression (2^-ΔΔCt)
(0.40)
(1.57)
(0.38)
(1.17)
(ns)
arnF expression (2^-ΔΔCt)
(0.70)
(1.45)
(0.38)
(1.28)
(ns)
pmrE/ugd expression (2^-ΔΔCt)
(0.40)
(1.82)
(0.42)
(1.18)
(ns)
pmrC expression (2^-ΔΔCt)
(0.50)
(2.52)
(0.30)
(1.66)
pmrA expression (2^-ΔΔCt)
(0.48)
(2.75)
(0.14)
(1.14)
(ns)
ΔcpxR/pMMB208
ΔcpxR/pCpxR-WT
ΔcpxR/pCpxR-Pneg

Supplementary Figure S6. Gene specific transcription is altered by modulation of CpxR levels in *Y. pseudotuberculosis*.

Quantitative RT-PCR was performed on mRNA isolated from *Y. pseudotuberculosis* cultured in LB at 26°C (A) or 37°C (B) until late logarithmic phase (*i.e.*: an incubation period of 5 hours with 10 uM IPTG). Data is a collation of six independent experiments composed of three technical replicates. Gene specific transcription of the 18 values was obtained from normalisation with the mean cycle threshold of two housekeeping genes, *gyrB* (n=18), and *rpoC* (n=18). After which three biological replicates of each target gene with both housekeeping standards were combined (6 data points) and are individually represented in a scatter plot as fold change relative to the calibrator control Δ*cpxR*/pMMB208 normalised to Log_2fold=1 after $2^{\Lambda-\Delta\Delta Ct}$ calculation. The median value from all data points is indicated by the bar height, and the actual fold difference in up- (>1.0) or down-regulation (<1.0) of specific gene expression relative to calibrator is indicated in parenthesis. Statistical analysis was performed using a two-tailed, unpaired t-test with Welch's correction where equal variance is not assumed, and confidence interval was set to 99%. Differences with a *P*-value of <0.05, < 0.01 or < 0.001 were considered significantly different from calibrator control and are indicated by a red single (*), double (**) or triple (***) asterisk situated immediately above the respective data points on the scatter plot. Analysed genes were: *lpxP* encoding for Palmitoleoyl acyltransferase (locus tag YPK_0430); *lpxL* encoding for Lauroyl acyltransferase (YPK_1665); *arnB/pmrH/L-ara4N* encoding for UDP-4-amino-4-deoxy-L-arabinose-oxoglutarate aminotransferase (YPK_1831); *arnF* encoding for Undecaprenyl phosphate-alpha-L-*ara4N* flippase subunit ArnF (YPK_1837); *pmrE/ugd* encoding for UDP glucose 6-dehydrogenase (YPK_2077); *pmrC* encoding for serine-type D-Ala-D-Ala carboxypeptidase/endopeptidase (penicillin-binding protein 4) (YPK_3740); *pmrA/basR* encoding for the two-component response regulator PmrA (YPK_3741). Strains: Δ*cpxR* null mutant/pMMB208 (empty vector); Δ*cpxR* null mutant/pCpxR$_{WT}$; Δ*cpxR* null mutant/pCpxR$_{Pneg}$.

Literature cited

1 Wolf-Watz, H., Portnoy, D. A., Bölin, I. & Falkow, S. Transfer of the virulence plasmid of Yersinia pestis to Yersinia pseudotuberculosis. *Infect Immun* **48**, 241, doi:10.1128/iai.48.1.241-243.1985 (1985).

2 Carlsson, K. E., Liu, J., Edqvist, P. J. & Francis, M. S. Extracytoplasmic-stress-responsive pathways modulate type III secretion in Yersinia pseudotuberculosis. *Infect Immun* **75**, 3913, doi:10.1128/iai.01346-06 (2007).

3 Liu, J., Obi, I. R., Thanikkal, E. J., Kieselbach, T. & Francis, M. S. Phosphorylated CpxR restricts production of the RovA global regulator in Yersinia pseudotuberculosis. *PLoS One* **6**, e23314, doi:10.1371/journal.pone.0023314 (2011).

4 Wang, R. F. & Kushner, S. R. Construction of versatile low-copy-number vectors for cloning, sequencing and gene expression in Escherichia coli. *Gene* **100**, 195 (1991).

5 Thanikkal, E. J. *et al.* The Yersinia pseudotuberculosis Cpx envelope stress system contributes to transcriptional activation of rovM. *Virulence* **10**, 37, doi:10.1080/21505594.2018.1556151 (2019).

6 Morales, V. M., Bäckman, A. & Bagdasarian, M. A series of wide-host-range low-copy-number vectors that allow direct screening for recombinants. *Gene* **97**, 39, doi:10.1016/0378-1119(91)90007-x (1991).

7 Gahlot, D. K., Wai, S. N., Erickson, D. L. & Francis, M. S. Cpx-signalling facilitates Hms-dependent biofilm formation by Yersinia pseudotuberculosis. *NPJ Biofilms Microbiomes* **8**, 13, doi:10.1038/s41522-022-00281-4 (2022).

8 Barbieri, C. M. & Stock, A. M. Universally applicable methods for monitoring response regulator aspartate phosphorylation both in vitro and in vivo using Phos-tag-based reagents. *Anal Biochem* **376**, 73 (2008).

9 De Wulf, P., McGuire, A. M., Liu, X. & Lin, E. C. Genome-wide profiling of promoter recognition by the two-component response regulator CpxR-P in Escherichia coli. *J Biol Chem* **277**, 26652, doi:10.1074/jbc.M203487200 (2002).

10 Yamamoto, K. & Ishihama, A. Characterization of copper-inducible promoters regulated by CpxA/CpxR in Escherichia coli. *Biosci Biotechnol Biochem* **70**, 1688, doi:10.1271/bbb.60024 (2006).
